# Pain-Associated Diagnoses in Childhood Before the Diagnosis of Attention-Deficit/Hyperactivity Disorder: A Population-Based Study

**DOI:** 10.3390/children11111388

**Published:** 2024-11-15

**Authors:** Eugene Merzon, Eli Magen, Yaniv Levy, Shai Ashkenazi, Iris Manor, Abraham Weizman, Beth Krone, Stephen V. Faraone, Ilan Green, Avivit Golan-Cohen, Shlomo Vinker, Ariel Israel

**Affiliations:** 1 Adelson School of Medicine, Ariel University, Ariel 4070000, Israel; shaias@ariel.ac.il; 2 Leumit Health Services, Tel-Aviv 6473817, Israel; allergologycom@gmail.com (E.M.); ylevi5@leumit.co.il (Y.L.); igreen@leumit.co.il (I.G.); agolanchoen@leumit.co.il (A.G.-C.); svinker@leumit.co.il (S.V.); aisrael@leumit.co.il (A.I.); 3 Medicine A Department, Assuta Ashdod Medical Center affiliated with the Ben Gurion, University of the Negev, Beer Sheva 8410501, Israel; 4 Faculty of Health Sciences, Ben Gurion University of the Negev, Beer Sheva 8410501, Israel; 5 Department of Family Medicine, Faculty of Medicine, Tel Aviv University, Tel Aviv 6997801, Israel; 6 Institute of Pain Medicine, Sheba Medical Center, Ramat Gan 5262000, Israel; 7 ADHD Unit, Geha Mental Health Center, Petah Tikva 491000, Israel; dr.iris.manor@gmail.com (I.M.); weizmana@gmail.com (A.W.); 8 Department of Psychiatry, Sackler Faculty of Medicine, Tel Aviv University, Tel Aviv 6997801, Israel; 9 Icahn School of Medicine at Mount Sinai, New York, NY 10029, USA; beth.krone@mssm.edu; 10 Departments of Psychiatry and Neuroscience and Physiology, SUNY Upstate Medical University, Syracuse, NY 13210, USA; sfaraone@childpsychresearch.org; 11 Department of Epidemiology and Disease Prevention, Faculty of Medicine, Tel Aviv University, Tel Aviv 6997801, Israel

**Keywords:** pediatric pain-associated diagnoses, ADHD, inflammation

## Abstract

**Background:** Pediatric pain significantly affects children’s lives, leading to school absenteeism, impaired social interactions, and psychological distress. The perception of sensory signals as pain is influenced by the brain’s noradrenergic system, and recent evidence suggests that chronic pain may impact cognitive functioning and emotional regulation. Attention-Deficit/Hyperactivity Disorder (ADHD) is associated with alterations in the dopaminergic/noradrenergic systems, which could affect pain perception. Pain-associated conditions and frequent analgesic use in childhood may be linked to ADHD development and could serve as early indicators, yet data on this potential association remain limited. **Study Aim:** This population-based case-control study in Israel aimed to assess the prevalence of pain-related diagnoses prior to ADHD diagnosis in children aged 5 to 18. The study included children registered with Leumit Health Services (LHS) between 1 January 2006, and 30 June 2021. Children diagnosed with ADHD were compared to matched controls, selected based on age, gender, socioeconomic status, and other sociodemographic factors, who were never diagnosed with ADHD during the study period. **Results**: Children with ADHD (*N* = 18,756) and controls (*N* = 37,512) were precisely matched for sociodemographic characteristics. Individuals with ADHD exhibited significantly higher frequencies of diverse pain conditions, including those associated with illness [headache, earaches, and throat pain (odds ratios [OR] = 1.156 [95%CI 1.085, 1.232], 1.295 [95%CI 1.217, 1.377], and 1.080 [95%CI 1.019, 1.145], respectively; *p* < 0.01)] and injury [sprains and strains (OR = 1.233 [95% CI 1.104,1.376)]. Analgesics were more frequently purchased by individuals with ADHD, particularly paracetamol (OR = 1.194 [95%CI 1.152, 1.237], *p* < 0.001) and ibuprofen (OR = 1.366 [95%CI 1.318, 1.416], *p* = 0.001). **Conclusions:** This study highlights a potential connection between ADHD and pediatric pain. The elevated rates of pain diagnoses and analgesic usage among children with ADHD underscore the need for further research.

## 1. Introduction

### 1.1. Pain-Associated Diagnoses in Children

Pain is a primary reason for seeking medical attention across all age groups, a phenomenon not exempting children [1]. A pediatric population-based survey revealed that 54% of the respondents had experienced pain within the previous three months [2]. Common pediatric pain-associated diagnoses encompass musculoskeletal pain, headaches, abdominal pain, and disease-related pain such as otalgia and throat pain [1,3,4,5,6,7]. Pain-associated diagnoses present a considerable burden on the healthcare system [8].

Musculoskeletal pain in children and adolescents is a common complaint. Its etiology was addressed in a study of over 400 pediatric patients [7]. Non-inflammatory and mechanical musculoskeletal pain accounted for 42% of the cases, with rheumatic, infectious, and malignant causes accounting for 31%, 21.6%, and 2.4%, respectively. The prevalence of musculoskeletal pain in the pediatric population can reach up to 40% in some studies [4] with considerable consequences [8].

Various types of headaches are notably prevalent in pediatric populations and may impact children differently based on age and individual characteristics. A recent meta-analysis found a pooled prevalence of 11% for migraines, 17% for tension-type headaches, and a pooled prevalence of 62% for overall primary headaches in children and adolescents (38% and 27% in females and males, respectively [9,10].

Otalgia may be caused by infectious entities, mainly acute otitis media and otitis externa, which accounts for most ear pain [11], or caused by dental pathologies, temporomandibular joint disorders, trauma, and neuralgic causes [9,11,12]. Likewise, throat pain is most often linked to infectious causes, mainly viral upper respiratory tract infections and bacterial tonsillopharyngitis. Abdominal pain is another common diagnosis in pediatric care. In a community-based study, 75% of middle school and high school students reported abdominal pain, whereas 21% found it severe enough to affect activities, and 8% visited a physician [13,14,15,16].

### 1.2. Attention-Deficit/Hyperactivity Disorder

Recent epidemiological studies revealed that several pain-associated conditions had been found to occur at higher rates among children with Attention-Deficit/Hyperactivity Disorder [10,12,14].

Attention-Deficit/Hyperactivity Disorder (ADHD) is a neurodevelopmental disorder characterized by impairment and core symptoms of inattention, hyperactivity, and impulsivity. It is the most common neurodevelopmental disorder affecting children, with a reported global prevalence around 5–7% in school-aged populations [17]. The prevalence of ADHD in Western countries varies between 9% and 15%, depending on the specific diagnostic criteria used and the population being studied [18,19]. The etiology of ADHD is multifactorial, encompassing genetic, environmental, and neurobiological factors. Several studies have indicated that ADHD might be associated with infections during childhood [20,21,22,23,24]. Early exposure to infections, particularly those affecting the central nervous system, has been suggested as a potential risk factor for the development of ADHD [21,23,25]. Young individuals diagnosed with ADHD exhibit elevated levels of inflammatory markers like interleukin (IL) 1-ß, IL-6, and Tumor Necrosis Factor-alpha (TNF-α); this elevation is a possible indicator of an association between ADHD and inflammation [26,27]. This was corroborated by a recent systematic review that showed a higher predilection for cytokine gene polymorphism (IL-6 and TNF alpha genes) and elevated serum cytokines in patients with ADHD versus controls [28].

### 1.3. Pain and Neurodevelopmental Disorders

There is an association between ADHD and noradrenergic functioning. The locus coeruleus, with its extensive network of noradrenergic neurons projecting upwards to many structures, such as the prefrontal cortex, acts as a central hub and is thought to regulate attention and impulse control. Likewise, these A6 adrenergic nuclei have extensive axonal projections to the spinal cord and are considered to play a major role in modulating chronic and persistent pain [29].

Emerging evidence further highlights the complex interaction between pain and neurodevelopmental disorders, notably ADHD. Mundal et al. have sampled adolescents and young adults at three time points during a 9-year longitudinal study and have shown a higher prevalence of chronic and multisite pain in adolescents and young adults with ADHD as compared to controls [30]. Kaplan and colleagues have recently identified several temporal risk factors for the development of multisite pain in children, amongst which were attention difficulties. However, no formal diagnosis of ADHD was established [31].

Multiple pain experiences might influence cognitive functioning, attention, and emotional regulation, thereby potentially contributing to the manifestation of ADHD symptoms. Conversely, the sensory sensitivities often seen in individuals with ADHD could amplify pain perception and the experience of pain-associated distress. Exploring this association could uncover shared risk factors and common neurobiological pathways and guide potential intervention points. We suggest that multiple pain conditions in childhood may serve as early indicators of ADHD.

### 1.4. Aims of Study

We aimed to explore comprehensively the prevalence of various pain-associated diagnoses (regardless of whether they are acute or chronic) in children who were later diagnosed as having ADHD diagnosis, with a comparison to a control group.

## 2. Methods

### 2.1. Study Design

**Figure 1:**
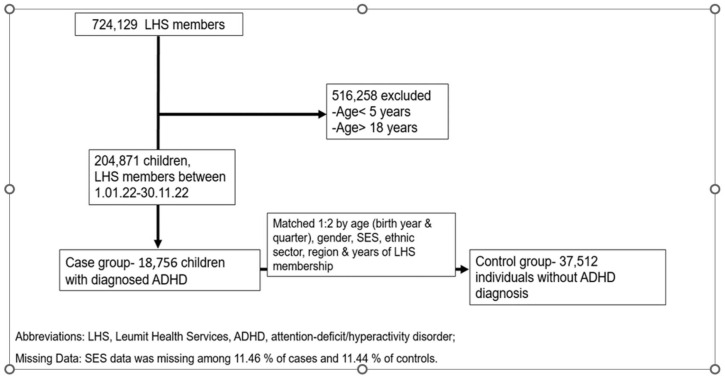
Study flowchart.

We conducted a population-based case-control study of ADHD-diagnosed children in a large Health Maintenance Organization (HMO) in Israel, Leumit Health Services (LHS), which served 724,129 persons during the study period. The information was collected from the HMO database. LHS maintains a comprehensive computerized database that is regularly updated with demographic information, medical visits, laboratory tests, hospitalizations, and medication prescriptions dating back to 1998. The database includes records of refilled and purchased prescriptions per patient. Diagnoses are recorded or updated during each physician visit according to the ICD-9 for somatic diagnoses and the ICD-10 for psychiatric diagnoses. This process ascertains the reliability of the diagnoses entered into the registry. The study design is shown in Figure 1.

### 2.2. Study Population

We included participants between 5 and 18 years old who were registered in LHS from 1 January 2006 to 30 June 2021. We excluded those with oncological disorders or immune deficiencies, as these conditions are rare and typically associated with unusually high rates of pain and infections. Cases were patients with an established diagnosis of ADHD. Controls were selected in a 2:1 ratio, randomly chosen from the study population. Control subjects were required to be free of any ADHD diagnosis prior to the date when their matched case received an ADHD diagnosis. Each subject in the control group was precisely matched to a case subject based on age, gender, ethnic sector (general population, Ultra-Orthodox Jews, and Israeli Arabs), socioeconomic status (SES) category, and year of initial LHS membership, to minimize potential confounding effects. Missing SES data (12.4%) were categorized separately for matching purposes. For each case, the 2 control subjects with the closest birth dates to the case’s birth date were selected. A post-hoc comparison of the main demographic variables was performed to verify similar distributions between the two groups (Table 1).

### 2.3. Definitions

ADHD cases were identified based on diagnoses that met the Israeli Ministry of Health criteria, which follow international diagnostic standards. The diagnosis had to be confirmed by a qualified senior physician—either a psychiatrist or neurologist (specializing in child or adult care), or a pediatrician or family physician with specialized ADHD certification. The diagnosis is established using the criteria outlined in the American Psychiatric Association’s Diagnostic and Statistical Manual (DSM-4 or 5, depending on the year of diagnosis). The validity of ADHD diagnoses was confirmed via a retrospective review of randomly selected electronic charts, with medical records thoroughly examined to ensure adherence to the Israeli Ministry of Health criteria and consistent documentation by certified ADHD specialists. SES was determined according to the classification of the Israeli Central Bureau of Statistics, which includes 20 subgroups. Classifications one to three are considered very low SES, four to six are low SES, seven to nine are medium SES, ten to fourteen are medium SES, and above fourteen is high SES.

### 2.4. Classification of Exposure

We defined two exposure categories: (1) any pain-associated diagnosis; and (2) using analgesic medications. The pain-associated diagnoses and analgesic medications use were considered as exposure only if these events occurred before the ADHD diagnosis.

### 2.5. Statistical Analysis

Statistical analysis was conducted using R-statistic software R 4.0.2 (R Foundation, Vienna, Austria), with two-sided tests and a significance level of 0.05. Sociodemographic characteristics between the ADHD and non-ADHD control groups were compared using the *t*-test and Fisher exact χ^2^ test for continuous and categorical variables based on the normal distribution and variable characteristics. The probabilities of having had pain-associated diagnoses among children with ADHD compared to the controls were compared using logistic regression analysis. The odds ratio (OR) and 95% confidence interval (CI) were calculated to demonstrate the effect sizes in this study. We used the Benjamini–Hochberg procedure to control the false discovery rate (FDR) for multiple tests.

### 2.6. Ethical Consideration

This study followed the Code of Ethics of the World Medical Association. The study was approved by institutional review committees of the LHS (approval number: LEU-0005-22). Due to this study’s retrospective and database nature, the need for informed consent was waived.

## 3. Results

### 3.1. Characteristics of the Study Groups

The ADHD case group included 18,756 subjects aged 5–18 years (mean 8.3 years, SD 2.6 years), and the 2:1 matched control group included 37,512 subjects. The sociodemographic attributes of the study cohorts are presented in Table 1. Across all variables, encompassing age distribution, age categories, gender, associated sector, and SES, a remarkable similarity was observed with no statistically significant distinctions. Many parameters were identical in the ADHD and control groups. This outcome underscores the effectiveness of the matching process.

### 3.2. Pain-Associated Diagnoses

The types and rates of pain-associated diagnoses in both the ADHD and control groups are detailed in Table 2. The frequency of pain diagnoses was notably higher in children with ADHD compared to the meticulously matched control group. The more prevalent pain diagnoses were not confined to a singular bodily system; instead, they encompassed diverse general pain conditions such as headache, otalgia, and throat pain (odds ratios (OR) and 95% confidence interval (95% CI): 1.156 [95% CI, 1.085, 1.232], 1.295 [95% CI, 1.217, 1.377] and 1.080 [95% CI, 1.019, 1.145], respectively, *p* < 0.01) as depicted in Table 2. Markedly elevated rates were found for all types of abdominal pain diagnoses, with an average effect size of 14% (*p* < 0.01 for all); for all types of limb pain diagnoses, with an average effect size of 35% (*p* < 0.01 for all), all types of arthralgias, with an average effect size of 40% (*p* < 0.001 for all), and diagnoses of sprains and strains of joints and adjacent muscles (OR 1.233, [95% CI 1.104, 1.376], *p* < 0.01).

### 3.3. Use of Analgesics

Table 3 provides insights into the specific categories of analgesic agents the study group utilized. The rates of analgesics purchased for individuals with ADHD were significantly higher compared to those prescribed to individuals without ADHD. The data for paracetamol use were OR = 1.194 (95% CI 1.152, 1.237), *p* < 0.001, and for ibuprofen use were OR = 1.366 (95% CI, 1.318, 1.416), *p* = 0.001, (details in Table 3).

## 4. Discussion

### 4.1. New Findings

In the present study, we investigated the prevalence of pain diagnoses in children who were later diagnosed with ADHD, relative to children who were never diagnosed with ADHD during the study timeframe.

Our findings demonstrate not only statistically significant associations between ADHD and a whole range of pain diagnoses, but also clinically meaningful effect sizes. Children diagnosed with ADHD had a 14% higher occurrence of prior abdominal pain diagnoses, a 35% higher occurrence of limb pain diagnoses, and a 40% higher occurrence of arthralgia compared to children without an ADHD diagnosis during the study period. These findings underscore the complex nature of the association between ADHD and physical health, with pain diagnoses spanning various physiological systems.

Our findings align with emerging evidence suggesting pain as an associated factor for ADHD [25].

Several theories have been proposed to clarify the interplay between ADHD and the elevated prevalence of pain conditions. As early as 2000, Anand KJ et al. postulated that neurodevelopmental changes to the immature perinatal brain could promote behavioral changes later in life. The proposed mechanism suggests that excitotoxic damage to developing neurons, mediated by excess NMDA/excitatory amino acid, e.g., as seen in exposure to repetitive pain, could lead to distinct behavior changes that include, among other attributes, altered pain sensitivity and ADHD, thus lending support to the neurodevelopmental shared basis theory of ADHD and pain [32].

Another forefront theory suggests neuroinflammation as a common denominator for ADHD and altered pain perception. Ample evidence suggests that maternal immune activation during pregnancy could affect the fetal brain and predispose toward neonatal neurodevelopmental disorders, and a putative mechanism was postulated [33,34]. In mice, maternal immune activation was shown to be associated with altered pain sensitivity [35].

Song et al. postulate a mechanism by which mast cell activation, which is considered fundamental in some pain-associated diagnoses, could be linked through neuromodulation and neuroinflammation to the development of ADHD [35]. While our study did not assess the bidirectional relationships between ADHD and pain conditions, evidence suggests that ADHD may increase susceptibility to pain. For instance, Jain et al. identified molecular interactions between nociceptors and immune cells that could heighten pain sensitivity in individuals with ADHD [36]. Kerekes et al. suggested that the altered pain perception in ADHD may be related to common neurobiological mechanisms, which involved dopaminergic systems underlying both ADHD and pain processing [26,37].

### 4.2. Strengths and Limitations

This study offers several strengths. Notably, it diligently matched cases and controls, ensuring a robust comparison. The design of a nationwide population-based survey enhances the generalizability of the findings. Including a large sample size provided ample statistical power for meaningful analyses.

Certain limitations warrant consideration. While our study relied on robust clinical records for ADHD diagnoses, based on the strict criteria set by the Israeli Ministry of Health, we acknowledge that a more in-depth evaluation of ADHD symptoms and their severity would require a detailed analysis of the text-based information contained within the electronic medical records. Additionally, the classification of controls solely based on diagnostic status leaves room for the possibility that some controls may have had undiagnosed ADHD, potentially influencing the study outcomes (albeit in a manner contrary to the reported findings). Furthermore, unmeasured confounding factors might contribute to the observed associations. For instance, parents of children with ADHD may have undiagnosed ADHD by themselves and seek medical care for their children due to general anxiety related to ADHD. One of the key limitations of this study is the absence of detailed information on whether the pain diagnoses were primary or secondary for each healthcare encounter, and the data do not allow for the determination of whether the pain conditions were acute or chronic. This could influence the observed associations, as chronic pain may have a more significant impact on child development. However, the mean age of study participants was approximately 8.3 years. At this age, the prevalence of chronic pain is typically lower than in older children and adolescents, making the distinction between acute and chronic pain less critical in the context of our findings. Future research incorporating detailed pain assessments and diagnostic criteria for chronic pain is needed to further explore this relationship.

Another important limitation is the lack of assessment of stimulant and non-stimulant medication use (which could potentially influence pain perception or reporting) among children with an ADHD diagnosis. Future targeted prospective studies could help to address these limitations and provide a more comprehensive understanding of the potential relationships between ADHD and pain.

## 5. Conclusions

The present study contributes valuable insights into the potential connection between ADHD and pain syndromes in childhood. The elevated rate of ADHD diagnosis among children having prior pain-associated diagnoses and analgesic prescriptions underscores the need for further research to elucidate the mechanisms driving this association and inform strategies for more holistic care for individuals facing both neurodevelopmental and somatic health challenges.

The current study focused on pain diagnoses preceding ADHD diagnosis, but the link between ADHD and pain disorders warrants further investigation. Prospective research is essential to comprehensively understand whether ADHD might also precede to chronic pain conditions.

## Figures and Tables

**Table 1:** Sociodemographic characteristics of the study population.

Characteristics	ADHD Cases N. (%)	MatchedControls N. (%)	OR	*p*-Value
No. of children	18,756	37,512		
Sex				
male	11,810 (63.0%)	23,619 (63.0%)	1.00	1
female	6946 (37.0%)	13,893 (37.0%)	1.00	1
Mean age of ADHD diagnosis, years, (SD)	8.3 (2.6)	8.3 (2.7)	-	0.99
Mean age of any pain diagnosis, years, (SD)	4.2 ± 1.5	4.1 ± 1.3	-	0.99
Sector				
Secular Jews	8402 (44.8%)	16,802 (44.8%)	1.00	0.99
Ultra-orthodox Jews	7737 (41.3%)	15,476 (41.3%)	1.00	0.99
Religious Jews	348 (1.9%)	696 (1.9%)	1.00	1
Arabs	2269 (12.1%)	4538 (12.1%)	1.00	1
SES, mean (SD)	15.3 (25.5)	14.5 (24.6)		0.09
Very low	5438 (14.5%)	2716 (14.5%)	1	1
Low	7980 (21.3%)	3990 (21.3%)	1	1
Medium	6456 (17.2%)	3221 (17.2%)	1	1
High	12,988 (34.6%)	6494 (34.6%)	1	1
Missing	4650 (12.4%)	2335 (12.4%)	1	1

**Table 2:** Diagnoses of pain syndromes in children with Attention-Deficit/Hyperactivity Disorder (ADHD) and controls.

ICD-9 Code—Diagnosis, Number (%)	ADHD Cases N = 1,875,699	Matched Controls N = 37,512	OR [CI]	*p*-Value	FDR BH
**784.0—Headache**	1653 (8.80%)	2894 (7.71%)	1.156 [1.085, 1.232]	0.0001	0.0001
**346—Migraine**	61 (0.32%)	80 (0.21%)	1.527 [1.075, 2.158]	0.0154	0.1523
**379.91—Eye Or Eye Region Pain**	75 (8.80%)	115 (0.31%)	1.306 [0.962, 1.763]	0.0763	0.4753
**388.7—Otalgia [Ear Pain]**	1853 (9.88%)	2928 (7.81%)	1.295 [1.217, 1.377]	0.0001	0.0001
**388.70—Otalgia, Unspecified [Ear Pain]**	367 (1.96%)	643 (1.71%)	1.144 [1.003, 1.305]	0.0433	0.3313
**388.71—Otogenic Pain**	98 (0.52%)	147 (0.39%)	1.335 [1.023, 1.737]	0.0296	0.2577
**784.1—Throat Pain**	1975 (10.53%)	3685 (9.82%)	1.080 [1.019, 1.145]	0.0088	0.1008
**789.00—Abdominal Pain, Unspecified Site**	2528 (13.48%)	4371 (11.65%)	1.181 [1.120, 1.245]	0.0001	0.0001
**789.07—Abdominal Pain, Generalized**	1175 (6.26%)	2117 (5.64%)	1.117 [1.037, 1.203]	0.0033	0.0458
**789.05—Abdominal Pain, Periumbilical**	696 (3.70%)	1266 (3.37%)	1.103 [1.003, 1.213]	0.0430	0.3298
**789.03—Abdominal Pain, Lower Quadrant**	442 (2.36%)	766 (2,04%)	1.158 [1.026, 1.305]	0.0162	0.1582
**729.58—Pain In Leg**	653 (3.48%)	1063 (2.83%)	1.237 [1.118, 1.367]	0.0001	0.0006
**729.56—Pain In Knee**	305 (1.63%)	468 (1.25%)	1.308 [1.128, 1.516]	0.0004	0.0063
**729.54—Pain In Hand**	174 (0.93%)	253 (0.67%)	1.379 [1.129, 1.681]	0.0014	0.0209
**729.57—Pain In Ankle**	105 (0.56%)	143 (0.38%)	1.471 [1.132, 1.907]	0.0030	0.0410
**719.460—Pain In Joint [Arthralgia] Involving Knee**	179 (0.95%)	279 (0.74%)	1.286 [1.059, 1.558]	0.0096	0.0108
**719.47—Pain In Joint [Arthralgia] Involving Ankle And Foot**	389 (2.07%)	664 (1.77%)	1.175 [1.033, 1.336]	0.0133	0.1366
**719.43—Pain In Joint [Arthralgia] Involving Forearm**	28 (0.15%)	32 (0.08%)	1.751 [1.016, 3.004]	0.0388	0.0302
**840–848—Sprains And Strains Of Joints And Adjacent Muscles**	539 (2.87%)	879 (2.34%)	1.233 [1.104, 1.376]	0.0002	0.0035

**Table 3:** Analgesics use in children with Attention-Deficit/Hyperactivity Disorder (ADHD) and controls.

Number (%)	ADHD Cases N = 18,756	Matched Controls N = 37,512	OR (CI)	*p*-Value	FDR BH
**N02—Analgesics**	9050 (48.25%)	16162 (43.08%)	1.232 [1.189, 1.276]	0.0001	0.0001
**N02B—Other Analgesics and Antipyretics**	9037 (48.18%)	16145 (43.04%)	1.231 [1.188, 1.275]	0.0001	0.0001
**N02BE01—Paracetamol**	8325 (44.39%)	15031 (40.07%)	1.194 [1.152, 1.237]	0.0001	0.0001
**M01AE01—Ibuprofen**	8282 (44.16%)	13752 (36.66%)	1.366 [1.318, 1.416]	0.0001	0.0001
**S02DA—Analgesics and Anesthetics**	3102 (16.54%)	5129 (13.67%)	1.251 [1.191, 1.314]	0.0001	0.0001
**N02BE51—Paracetamol, Combinations Excl. Psycholeptics**	947 (5.05%)	1539 (4.10%)	1.243 [1.143, 1.351]	0.0001	0.0001
**M02AA15—Diclofenac**	421 (2.24%)	702 (1.87%)	1.204 [1.063, 1.362]	0.0032	0.0172
**M01AE02—Naproxen**	254 (1.35%)	356 (0.95%)	1.433 [1.214, 1.690]	0.0001	0.0002
**S02DA30—Anesthetic Ear Drops**	1369 (7.30%)	2206 (5.88%)	1.260 [1.174, 1.352]	0.0001	0.0001
**D04AB—Anesthetics For Topical Use**	257 (1.37%)	436 (1.16%)	1.181 [1.008, 1.383]	0.0385	0.1105
**M02AA—Anti-inflammatory Preparations, Non-steroids For Topical Use**	469 (2.50%)	790 (2.11%)	1.192 [1.060, 1.340]	0.0030	0.0013
**M02—Topical Products For Joint and Muscular Pain**	670 (3.57%)	1127 (3.00%)	1.196 [1.083, 1.319]	0.0004	0.0007
**28612—Elastic Bandage 10 cm**	68 (0.36%)	81 (0.22%)	1.682 [1.199, 2.351]	0.0022	0.0303

## Data Availability

The data presented in this study are available upon request and with restrictions from Leumit Health Services due to privacy and confidentiality concerns. Access to the data requires authorization in compliance with institutional policies and relevant data protection regulations to ensure patient privacy.
